# Supplementary material for: Interlayer quantum transport in Dirac semimetal BaGa2
Source: Nat Commun. 2020 May 12;11:2370. doi: 10.1038/s41467-020-15854-0 (PMC7217856; doi:10.1038/s41467-020-15854-0)
Supplement: Supplementary file 1 — Supplementary Information [file 41467_2020_15854_MOESM1_ESM.pdf]

Supplementary Materials

Interlayer quantum transport in Dirac semimetal $BaGa_2$

Xu *et al*.

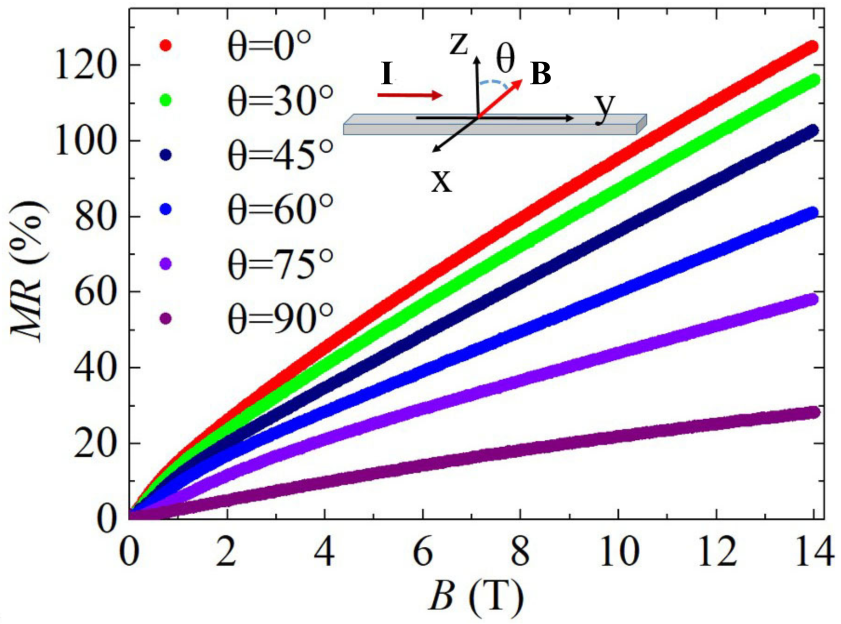

Supplementary Figure 1. Field dependence of in-plane MR at different angles and 2.5 K.

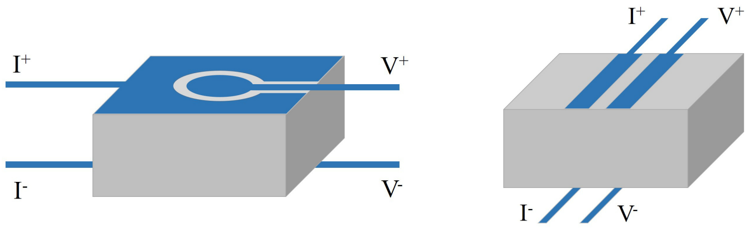

Supplementary Figure 2. Schematic drawing of setup for interlayer transport measurement.

Supplementary Table I. Landau level index N for the trivial pockets. These parameters are obtained from the dHvA quantum oscillation measurement at 14 T, which clearly shows that the system is far away from quantum limit.

	F_α	F_β	F_γ	F_η
LL index N	3	5	28	134
